# Stiripentol in Dravet syndrome: preventing prolonged seizures, reducing mortality, and improving value in care

**DOI:** 10.1055/s-0046-1829156

**Published:** 2026-10-27

**Authors:** Kette Valente, Roberto Caraballo

**Affiliations:** 1 Universidade de São Paulo, Faculdade de Medicina, Departamento de PsiquiatriaSão Paulo SPBrazil.; 2 Universidade de São Paulo, Faculdade de Medicina, Centro Integrado de Doenças Genéticas e RarasSão Paulo SPBrazil.; 3 Hospital de Pediatria Prof. Dr. Juan P GarrahanBuenos Aires BsAsArgentina.

**Keywords:** Epilepsy, Myoclonic, Encephalopathy, Disease Burden, Anticonvulsants

## Abstract

Dravet syndrome (DS) is a rare and severe developmental and epileptic encephalopathy that begins in infancy. Although nonconvulsive seizures may occur, the syndrome is marked by frequent, prolonged *convulsive* seizures followed by neurodevelopmental plateau, slowing, or regression. Stiripentol (STP) is an anti-seizure medication (ASM) approved for adjunctive use in DS in countries across Europe, North America, Asia, South America, Oceania, and Africa, though it is not yet approved in most countries in Latin America. The present narrative review summarizes current evidence on the clinical efficacy, safety, and cost-effectiveness of (STP). Data from randomized controlled trials, real-world studies, and systematic literature reviews demonstrate that (STP) reduces seizure frequency and duration, increases seizure-free intervals, and helps prevent or interrupt status epilepticus (SE). These effects are particularly relevant for lowering the risk of premature mortality and improving the quality of life (QoL) for patients and caregivers. Stiripentol has also shown a consistent safety profile, including in patients treated for over 18 years. Pharmacoeconomic analyses indicate that (STP) achieves meaningful seizure reduction and seizure freedom at a lower cost compared with cannabidiol and fenfluramine, which are ASMs also approved for the treatment of DS. Cost-utility models further support (STP)'s value under commonly accepted willingness-to-pay thresholds. Taken together, current evidence supports the role of (STP) as an effective, well-tolerated, and economically favorable treatment option for individuals with DS, including from early infancy.

## DRAVET SYNDROME: A PROGRESSIVE DISORDER AND ITS MODIFIABLE FACTORS

### Defining Dravet syndrome as a progressive deterioration disorder

Dravet syndrome (DS) is a genetic, drug-resistant, and severe form of developmental and epileptic encephalopathy characterized by early-onset seizures, often between 3 and 9 months of age. 1 Most cases of DS are due to pathogenic variants in the voltage-gated sodium channel 1a ( *SCN1A* ) gene that result in haploinsufficiency of Nav1.1, the α‐1 subunit of the sodium channel. 2

Despite its rarity, affecting an estimated 1.2 to 6.5 individuals per 100 thousand (2,500–13,800 individuals in Brazil, based on the 2024 population of 212 million), 3 DS is associated with a profound lifelong burden. Seizure types in DS are heterogeneous and may include myoclonic, generalized tonic-clonic, focal impaired awareness, absence, atonic, and tonic seizures, with myoclonic and focal impaired awareness seizures being among the most frequently reported in early childhood. 4 Many patients experience daily or multiple daily seizures, averaging 23 per month, with some cases exceeding 100 monthly. 5 Another component of the DS phenotype is the presence of prolonged (lasting more than 5 minutes) *convulsive* seizures, often requiring emergency attendance at hospitals. 6 Nonconvulsive (obtundation) status epilepticus (SE) is also a recognized manifestation of DS 4 and has been associated with episodes of behavioral change and neurodevelopmental decline. While a causal relationship has not been definitively established, the temporal overlap suggests that nonconvulsive SE may contribute to or exacerbate cognitive and behavioral impairments in some patients. 7

Dravet syndrome rapidly progresses to a highly debilitating condition. From as early as 2 years of age, affected children begin to show neurodevelopmental impairments, including intellectual disability, speech impairment, sleep disturbances, motor deficits, and behavioral and psychosocial challenges. 1 6

### Quality of life in DS: humanistic impact

As a consequence of the high seizure burden and associated comorbidities, children with DS have significantly lower health-related quality of life (QoL) compared with both the general pediatric population and children with other seizure types, epilepsy types, and epilepsy syndromes. 8 Scores on the Pediatric Quality of Life Inventory highlight this disparity: children with DS scored 46.85 versus 84.61 in the general pediatric population (a 45% reduction) 8 and 54.7 versus 93.5 in children with other epilepsies (a 41% reduction). 9 This substantial impairment in QoL persists into adulthood, as shown by a 50% reduction in the EuroQol 5-Dimension 5-Level instrument (EQ-5D-5 L) utility scores in patients of all ages with DS (≥ 2 years old) compared with the general adult population. 10

Importantly, the DS burden is not limited to patients. Caregivers often provide constant supervision, leading to high rates of unemployment (28–44%), part-time work (29%), and absenteeism (40–65%). 6 Beyond the professional impact, caregiving substantially affects time, energy, sleep, social life, and family dynamics. 6 Not surprisingly, up to 70% of caregivers report symptoms of depression, 6 highlighting the emotional and mental health challenges faced by families of individuals with DS. Although specific data on caregivers of individuals with DS are lacking in Brazil, findings from the Americas Health Foundation 11 shed light on the broader reality of rare disease caregiving in the region. In Brazil, 75% of employed caregivers of people with rare diseases reported reduced willingness to work, 63% missed at least one day of work per month due to caregiving duties, and 61% expressed dissatisfaction with their job performance. Nearly all (97%) reported being preoccupied with the patient during working hours.

### The economic burden of DS

Dravet syndrome also imposes a substantial economic burden on healthcare systems and society. Informal care alone (often provided by family members) accounts for over 3,000 hours per year (equivalent of 8.5 hours every day). In a survey conducted in Colorado (US), the estimated annual cost of this care exceeded $80,000 when considering lost productivity and reduced leisure time. 12 In Germany, the annual healthcare costs for a patient with DS ranged between €11,048 and €24,172 in 2015 to 2017, 13 14 15 16 while in the US, costs ranged from $27,000 to $77,914 per patient per year. 12 17 18 19 Inpatient admissions are a key direct cost driver, representing 18 to 47% of total direct costs. 6 These elevated costs are closely linked to the severity of the disease. Epilepsy specialist visits, emergency department admissions, and ambulance use are 2.2, 1.8, and 2.8 times more frequent, respectively, in the highest seizure burden subgroup (composite seizure frequency [CSF] score 14–39) compared with those in the lowest seizure frequency group (CSF score 0–2). 16 Additionally, the frequency and duration of hospitalizations are lower among patients who have been prescribed anti-seizure medications (ASMs) compared with those who have not. 13 While comparable data are not available for Brazil, this highlights the potential magnitude of the economic burden also faced by Brazilian families.

### Premature mortality and modifiable factors

Overall, the mortality rate in DS is nearly 10 times higher than in matched individuals without DS. 13 Death occurs during childhood in 15 to 20% of patients, with a median age at death between 4.7 and 7.0 years. 3 20 21 Sudden unexpected death in epilepsy (SUDEP) and SE are the first and second leading causes of death in DS, respectively. 20 The Dravet-specific estimated SUDEP rate ranges from 4.4 to 9.32 per 1 thousand person-years. 21 22 The particularly high rates of SUDEP in DS are a consequence of multiple and interactive mechanisms, including the disruption of sodium channel function in both neurons and cardiomyocytes, which predisposes to peri-ictal arrhythmias such as bradycardia and asystole. 23 Seizure-related respiratory dysfunction, including ictal and postictal central apnea, impaired arousal, and brainstem serotonergic dysfunction, has also been identified as a major contributor to the risk of SUDEP, resembling mechanisms implicated in sudden infant death syndrome. 24 25 Moreover, postictal generalized electroencephalogram (EEG) suppression, which reflects a transient global depression of cortical and brainstem activity after generalized tonic-clonic seizures (GTCS), has been associated with increased SUDEP risk. 26

Recurrent GTCS and frequent SE, highly prevalent in DS, further amplify this risk of premature mortality. 27 Convulsive SE is not only a hallmark of DS, but it is also an encephalopathic driver, contributing to acute morbidity and long-term developmental decline. Fatal outcomes are often precipitated by febrile or refractory SE, 27 28 and repeated episodes have been associated with regression in adaptive functioning and worsening neurocognitive outcomes over time. 29

## STANDARD OF CARE IN DS: PREVENTING MORTALITY AND MINIMIZING NEURODEVELOPMENTAL IMPAIRMENT

Managing DS remains particularly challenging due to its well-documented resistance to conventional ASMs. Patients often require multiple medications, and some may try up to 12 different ASMs in search of seizure control. 30

Effective management of DS aims to control convulsive seizures (particularly seizure clusters and SE), reduce the overall frequency of seizures, and minimize adverse effects of ASMs, with the ultimate objective of improving QoL and lowering the risk of premature death due to SE and SUDEP, as these events represent the most acute threat to both life and long-term development. 4 Figure 1 illustrates the current standard of care for DS, highlighting the recommended role of stiripentol (STP) as a first- or second-line adjunctive therapy. 31 32 33 34

**Figure 1:**
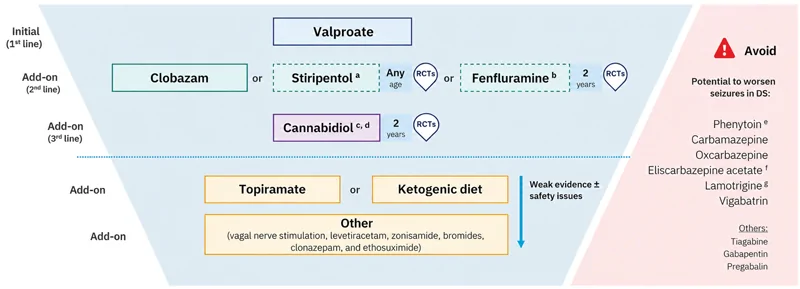
Abbreviations: DS, Dravet syndrome; RCT, DS-specific therapy supported by randomized clinical trial evidence. Notes: ^a^ With clobazam and valproate (US: clobazam); ^b^ EU: add-on to other anti-epileptic medicines; ^c^ EU: in conjunction with clobazam; ^d^ In Brazil, the approved cannabidiol formulation contains 0.2% tetrahydrocannabinol and was not approved based on RCTs; ^e^ Phenytoin is used in SE; ^f^ Not available in Brazil; ^g^ Coadministration with valproate is possible but requires caution, as it may exacerbate myoclonic seizures. Source: Adapted from Wirrell et al. (2022). 4 Current standard of care for DS. The internationally recommended standard of care follows a stepwise add-on approach starting with valproate as initial therapy, followed by clobazam, stiripentol and fenfluramine as second-line add-on agents, and cannabidiol as third-line add-on drug. Stiripentol is the only DS-specific treatment approved for use in infants as young as 6 months of age in the United States, and with no minimum age restriction in several other countries. Fenfluramine and cannabidiol are approved from age 2 years. Topiramate, ketogenic diet, and other options may be considered in later lines, although supported by limited evidence. Sodium channel blockers are contraindicated due to seizure aggravation and worse developmental outcomes. Medications in dashed-line boxes are not currently available in Brazil.

Stiripentol represents a cornerstone in the pharmacological management of DS, particularly as part of the canonical regimen in combination with valproate and clobazam. 31 32 33 34 Beyond its proven efficacy in reducing overall seizure burden, randomized controlled trials and subsequent real-world studies have demonstrated that STP significantly decreases the incidence of prolonged seizures and SE—key drivers of morbidity and mortality in DS. 35 36

The clinical value of STP is further reinforced by recent systematic reviews and meta-analyses, which confirm that it provides meaningful reductions in GTCS and contributes to lowering the risk of seizure-related emergencies requiring hospitalization. 37 38 Given that recurrent convulsive SE and poorly controlled GTCS are the most important risk factors for SUDEP in DS, 20 39 the clinical relevance of STP may extend beyond seizure frequency reduction alone. In DS, these severe motor events act as critical encephalopathic drivers, linked not only to mortality-related outcomes, but also to acute neurological deterioration and cumulative neurodevelopmental burden. 4 Therefore, by reducing the occurrence of prolonged convulsive seizures and SE, STP may help lower both the immediate risk of seizure-related emergencies and the longer-term functional impact associated with recurrent convulsive insults. This hypothesis is supported by preclinical evidence. In mouse models of SUDEP based on audiogenic seizures, STP dose-dependently prevented seizures, and seizure-induced mortality when administered systemically before audiogenic stimulation. 40 Similarly, in a methionine sulfoximine-induced SE mouse model, STP significantly reduced the incidence of SE, seizure burden, and mortality in a dose-dependent manner. 41

In light of these findings, optimizing access to STP is a critical therapeutic and public health priority, particularly in regions where the drug remains unavailable or not reimbursed. 31 Notably, STP is the only DS-specific treatment approved for use in infants as young as 6 months of age in the US, and with no minimum age restriction in several other countries. Since its initial approval in Europe in 2007, it has been prescribed in 55 countries, accumulating more than 69,000 patient-years of exposure in the treatment of DS. 42

## MECHANISMS OF ACTION OF STP

Stiripentol has been shown to exert its anti-seizure effects in DS through multiple mechanisms, as illustrated in Figure 2 . Its primary mode of action involves enhancement of gamma-aminobutyric acid (GABA)-mediated neurotransmission. This effect is achieved through several complementary pathways: inhibition of GABA reuptake, reduced enzymatic degradation of GABA, and positive allosteric modulation of GABA _A_ -type receptors. GABA _A_ receptor is a heteropentamer, in which GABA binds at the α/β subunit interfaces, while benzodiazepines act at the α/γ interface. 43 Stiripentol modulates distinct sites, particularly those containing α3 and δ subunits, 43 which explains its non-competitive action relative to benzodiazepines. Importantly, STP binds to GABA _A_ receptor at synaptic and extrasynaptic localizations, leading to both phasic and tonic inhibitions. 43 These mechanisms of STP collectively enhance inhibitory signaling in the central nervous system, counteracting the neuronal hyperexcitability characteristic of DS. In addition to its effects on GABAergic transmission, STP also modulates ion channel activity, particularly voltage-gated sodium channels and T-type calcium channels, 43 44 both of which are known to contribute to the generation and propagation of epileptic discharges. 43 Notably, T-type calcium channel blockade may reduce calcium influx associated with excitotoxicity, suggesting a potential neuroprotective effect of STP. 43 45 Moreover, by enhancing GABAergic transmission and inhibiting CYP-mediated metabolism of clobazam and valproate, STP potentiates their anti-seizure effects, achieving more sustained seizure control. 35

**Figure 2:**
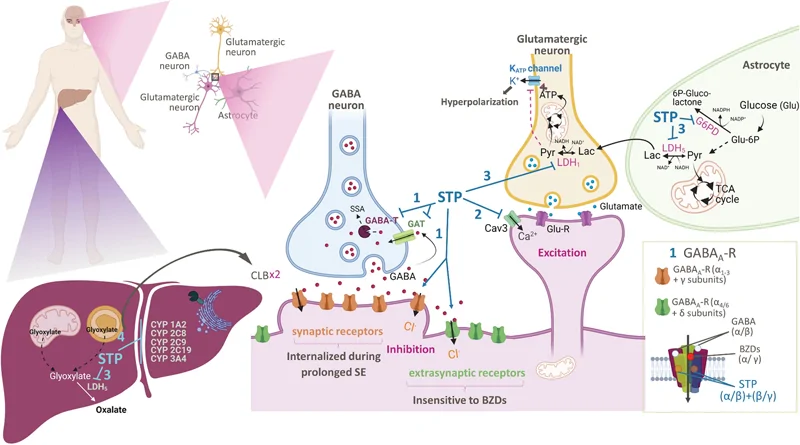
Abbreviations: BZDs, benzodiazepines; ATP, adenosine triphosphate; NAD, nicotinamide adenine dinucleotide; NADP, nicotinamide adenine dinucleotide phosphate; TCA, tricarboxylic acid. Source: Figure from Bacq et al. (2024) 43 ; Creative Commons license: http:// creativecommons.org/licenses/by-nc/4.0/. Summary of the different mechanisms of action of stiripentol (STP). 1) Stiripentol potentiates GABAergic transmission by reducing GABA degradation (inhibition of GABA transaminase, GABA-T), inhibiting GABA reuptake from the synaptic cleft by interacting with GABA transporter (GAT), and acting as a positive allosteric modulator of GABA _A_ receptors (GABA _A_ -R). STP can target both synaptic and extrasynaptic receptors, likely by binding of transmembrane subunits (bottom right). 2) STP inhibits ion channels, such as postsynaptic T-type calcium channels: Cav3.1, 3.2, and 3.3. 3) STP can alter brain energy metabolism by inhibiting glucose-6-phosphate dehydrogenase (G6PD) and lactate dehydrogenase (LDH), involved in lactate (Lac)-pyruvate (Pyr) shuttle. In the liver, STP also inhibits LDH5, which is responsible for oxalate production. 4) Stiripentol makes pharmacokinetic interactions with concomitant anti-seizure medications, such as clobazam (CLB), by inhibiting several cytochrome 450 (CYP) enzymes.

Emerging evidence also points to a role for STP in regulating cerebral energy metabolism. Preclinical studies have shown that STP modulates key enzymes involved in glucose and lactate pathways, including the inhibition of glucose-6-phosphate dehydrogenase (G6PD) and lactate dehydrogenase (LDH). 43 46 By reducing G6PD activity, STP may help prevent excessive glucose consumption in the brain during convulsive states, a phenomenon described as a “neuro/glial glucose-sparing effect.” 43 Additionally, LDH plays a central role in the astrocyte-neuron lactate shuttle, 47 a key pathway for energy transfer in the brain. Stiripentol has demonstrated inhibitory effects on both LDH1 and LDH5 isoenzymes in vitro. 48 Although these effects were observed at concentrations higher than those achieved in clinical settings, this suggests a potential mechanism to reduce seizure-associated lactate accumulation and preserve cellular energy homeostasis. These findings are in line with the growing recognition that disturbances in brain energy metabolism contribute to epileptogenesis. 49 Notably, STP's effects on LDH activity have also been observed in models of glioblastoma, 50 angiogenesis, 51 and cerebral ischemia. 52 Altogether, these data suggest that STP may exert broader neurobiological effects beyond seizure control, potentially contributing to neuroprotection and metabolic stabilization in epilepsy.

## CLINICAL AND HUMANISTIC VALUE OF STP

### Clinical efficacy

The efficacy of STP in DS has been robustly demonstrated in two pivotal, double-blinded, randomized, placebo-controlled phase-3 trials (Stiripentol in Severe Myoclonic Epilepsy in Infancy [STICLO]-France 35 and STICLO-Italy 53 ) followed by a post-hoc pooled analysis that incorporated additional outcomes of clinical relevance 5 ( Table 1 ). In both trials, STP was administered as an add-on to valproate (maximum dose 30 mg/kg/day) and clobazam (maximum dose 0.5 mg/kg/day) at a fixed dose of 50 mg/kg/day for 2 months, with no titration. Eligible patients were aged 3 to 18 years and had a diagnosis consistent with the clinical criteria for DS, including ≥ 4 GTCS per month despite optimized background therapy. Importantly, patients receiving other ASMs, except progabide and rectal diazepam in specific situations, were excluded, increasing the reliability of the findings by minimizing potential pharmacokinetic interactions with other ASMs. After the double-blind phase, participants entered a 1-month open-label extension in which all received STP. The post-hoc pooled analysis included 64 patients (33 on STP, 31 on placebo). At the end of the double-blind period, STP produced significantly greater reductions in monthly GTCS frequency compared with placebo (− 66% versus + 4%; *p* < 0.001). A clinically meaningful reduction in the frequency of GTCS (≥ 50%) was observed in 72% of patients in the STP group versus 7% in the placebo group. Moreover, 56% of STP-treated patients achieved ≥ 75% GTCS frequency reduction (versus 3% in the placebo group), and 38% were seizure-free, while none of the placebo recipients reached this threshold ( *p* < 0.001 for all comparisons). 5

**Table 1:** Efficacy and safety outcomes from randomized controlled trials of stiripentol in Dravet syndrome. Responder rates were based on reductions in clonic or tonic-clonic seizure frequency during the double-blind phase of each study. Stiripentol was administered at a fixed dose of 50 mg/kg/day in combination with valproic acid and clobazam. Placebo was also given as an add-on to valproic acid and clobazam. Data are presented as percentages and absolute numbers (n/N)

Study	Treatment arm	n	≥ 50% reduction in convulsive seizures	≥ 75% reduction in convulsive seizures	Seizure-free	Serious adverse events	Discontinuations due to adverse events
STICLO-France 35	Stiripentol	21	71.5% (15/21)	57.1% (12/21)	42.9% (9/21)	9.5% (2/21)	4.8% (1/21)
	Placebo	20	5% (1/20)	0% (0/20)	0% (0/20)	15% (3/20)	20% (4/20)
STICLO-Italy 53	Stiripentol	12	66.7% (8/12)	50% (6/12)	25% (3/12)	0% (0/12)	8.3% (1/12)
	Placebo	11	9.1% (1/11)	9.1% (1/11)	0% (0/11)	0% (0/11)	18.2% (2/11)
Pooled analysis 5	Stiripentol	33	72% (24/33)	56% (18/33)	38% (13/33)	6% (2/33)	Not Reported
	Placebo	31	7% (2/31)	3% (1/31)	0% (0/31)	9.6% (3/31)	Not Reported

Diary-based analyses further supported STP's benefits. The median total number of seizure-free days over 60 days was significantly higher in the STP group compared with placebo (55.8 versus 40.9 days; *p* < 0.001). Additionally, patients receiving STP experienced longer uninterrupted periods without seizures, with a median of 32 consecutive seizure-free days versus 8.5 days in the placebo group ( *p* < 0.001). 5 Stiripentol also demonstrated a rapid onset of action, with significant seizure reduction emerging by day 4 and persisting throughout the double-blind phase. Notably, patients who switched from placebo to STP during the open-label extension experienced an 80% reduction in seizure frequency, while efficacy was sustained in those already receiving the drug. 5

The extended seizure-free intervals observed after the treatment with STP may help reduce the risk of SUDEP, which accounts for nearly half of all deaths in DS and is closely linked to frequent convulsive seizures. 20 39 Sveinsson et al. 39 showed that patients who experience 1 to 3 GTCS per year have a 22-fold increased risk of SUDEP, while those who experience 4 to 10 GTCS per year face a 32-fold increase. Beyond the clinical benefits, seizure-free days also lead to measurable gains in QoL, with positive impacts observed in both patients and carers. Each additional seizure-free day in a 28-day period is estimated to improve utility scores by 0.005 for patients and 0.014 for carers ( *p* < 0.001). 54

In addition to its role in reducing seizure frequency, STP may also help prevent and interrupt SE, one of the most severe and life-threatening complications in DS. 20 In a systematic review of 15 human studies involving 474 patients aged 1.1 to 78 years, STP was associated with a ≥ 50% reduction in SE episodes in an average of 68% of patients (range: 41–100%). Moreover, between 26 and 100% of patients (mean: 77%) achieved complete freedom from SE after the initiation of STP. 55

Additional support for the use of STP comes from a large, real-world retrospective study involving 131 patients with DS who initiated treatment before 2 years of age. 56 Most patients (93%) received STP in combination with valproate and clobazam, at a median starting age of 13 months and a median dose of 50 mg/kg/day. In the short term (median 4 months on treatment), the frequency of tonic-clonic seizures lasting more than 5 minutes significantly declined ( *p* < 0.01) Moreover, SE resolved in 55% of patients. With longer follow-up (median 28 months), 67% of patients remained free of SEs and 62% achieved freedom from seizures > 5 minutes. These improvements are unlikely to reflect the natural course of DS, especially considering that in placebo-controlled trials, no patients receiving placebo achieved seizure freedom and/or remained free of SE. 35 57 In addition, these improvements were accompanied by a decline in emergency hospitalizations, from 91% at baseline to 43% after short-term treatment and 12% after long-term use ( *p* < 0.001). Notably, seizures lasting more than 5 minutes are nearly universal among children with DS, with 77% experiencing an episode by 1.5 years of age. 57 The ability of STP to reduce both the frequency and duration of seizures has also been consistently demonstrated in other real-world studies, as summarized in Table 2 . This effect, particularly when treatment is initiated early in life, highlights its relevance in mitigating high-risk outcomes such as SE and SUDEP. 20

**Table 2:** Summary of real-world evidence on the effectiveness of stiripentol in Dravet syndrome

Study	Population	Country	Median age at stiripentol initiation	Key findings
Inoue et al. (2009) 73	112 patients with DS	Japan	Not reported	After 4 weeks of treatment (early period), 61% of patients achieved more than 50% seizure reduction, and 8.7% remained seizure-free. After a mean of 14.1 months of treatment (late period), 48% of patients achieved more than 50% seizure reduction. Seizures were shortened in 43% (early period) or 22% (late period) of the patients. Caregivers reported improvement of behavior in 17% (early period) or 22% (late period) of the patients.
Wirrell et al. (2013) 78	82 patients with DS	United States	6.9 years	After a median of 22.3 months of treatment, overall seizure frequency was reduced in 2/6 patients treated with stiripentol without clobazam or valproate, 28/35 treated with stiripentol with clobazam but without valproate, 8/14 receiving stiripentol with valproate but without clobazam, and 30/48 treated with stiripentol with clobazam and valproate. All children with frequent prolonged seizures at baseline showed reduced frequency with stiripentol treatment.
Inoue et al. (2015) 77	24 patients with DS	Japan	Not reported (range 1–24 years)	After 56 weeks of treatment, 54% of the patients were classified as responders (seizures became ≤ 50% frequent as compared with baseline), with 2 patients remaining seizure-free.
Chiron et al. (2018) 65	40 patients with DS	France	6.3 years	Efficacy was maintained after a median of 18 years on stiripentol and up to 40 years of age. On stiripentol from childhood, 25% of Dravet patients exhibited > 1-year seizure-free periods during adulthood, and none had SE after 25 years of age.
Myers et al. (2018) 63	41 patients with DS	Australia and the United Kingdom	5.6 years (11 months–22 years).	Stiripentol improved long-term seizure frequency in ~ 50% of patients when used as part of unrestricted polytherapy. In 42% patients, episodes of SE decreased by 50% or more following stiripentol initiation.
Yıldız et al. (2019) 76	21 patients with DS	Turkey	Not reported	After a mean follow-up period of 42 months, the seizure frequency decreased by more than 50% in 12 patients (57%), and 2 of them were seizure-free. SE was not recorded after stiripentol treatment in 8 of 11 patients who experienced SE during the baseline period.
Yamada et al. (2021) 75	411 patients with DS	Japan	7 years	Convulsive seizures became ≤ 50% frequent as compared with baseline in 46.2%, 40.5%, and 42.8% of the patients after 16, 52, and 101 weeks on stiripentol, respectively. Overall improvement was rated as marked in 18.7% and moderate in 26.6% of new patients after 104 weeks or at the time of drug discontinuation. Symptoms worsened in only 5.1% of the patients.
Balestrini et al. (2022) 86	196 patients with DS with and non‐Dravet refractory developmental and epileptic encephalopathies	Italy		After a median follow-up of 3 months, 53% of patients responded and 9% achieved seizure freedom. Response rates declined to 29% at 12 months and 22% at 24 months. Sustained response was higher in DS (64% at 48 months) compared with other genetic (13%) or unknown causes of epilepsy (38%). Higher responder rates were also seen in patients with generalized (44%) or combined focal/generalized epilepsy (28%) versus focal epilepsy (20%). Relapse-free survival was greatest when stiripentol was started between 0–4 years or in adulthood.
Chiron et al. (2023) 56	131 patients with DS	France	12.6 months	With short-term therapy (< 6 months on stiripentol), the frequency of seizures lasting > 5 minutes decreased, and SE disappeared in 55% of patients. With long-term therapy (median 28 months), the frequency of seizures continued to decline. Emergency hospitalizations dropped from 91% to 43% and 12% with short- and long-term therapies, respectively.
Gil‐Nagel et al. (2024) 87	82 patients (55 with DS and 27 with non‐Dravet refractory developmental and epileptic encephalopathies)	Madrid	DS: 52.5 months (range 32.2–118) Non-DS: 114 months (49.8–183)	At 12 months, retention rate, responder rate and seizure free rate was 68.3%, 65% and 18%, respectively. There were no statistically significant differences between groups on these seizure outcomes.
Kitamura et al. (2025) 79	515 patients with DS	Japan	7 years	The rate of overall improvement (marked or moderate) after 156 weeks or at treatment discontinuation was 37.7%. In newly treated patients, seizure frequency decreased over 156 weeks across all seizure types, compared with the early phase of treatment.

Evidence from a network meta-analysis 58 reinforces the clinical value of STP in the treatment of DS. When used as a first-line add-on therapy, STP demonstrated superior seizure control compared with cannabidiol. Specifically, a reduction in monthly convulsive seizure frequency higher than 50% was achieved by significantly more patients treated with STP than with cannabidiol 10 mg (risk difference: 45%) or 20 mg (risk difference: 39%), with statistical significance in both comparisons ( *p* < 0.005). Although the difference in comparison to fenfluramine was not statistically significant in this category (1%; *p* = 0.93), STP was consistently associated with higher rates of complete seizure freedom (100% reduction), showing risk differences of 33% (versus cannabidiol 10 mg), 31% (versus cannabidiol 20 mg), and 26% (versus fenfluramine), all statistically significant ( *p* < 0.005). These findings, consistent with those of previous meta-analyses, 37 38 59 60 61 suggest that STP may offer clinically meaningful seizure reduction and a higher likelihood of complete seizure freedom compared with DS-specific treatments developed after STP.

Although most clinical evidence supporting STP's efficacy comes from studies in which it was administered alongside valproate and clobazam, real-world experience indicates it is beneficial even in the absence of clobazam or valproate. 62 63 This is particularly relevant in Latin America, where the availability of DS-specific medications can be inconsistent and shortages occasionally occur. 64 In such contexts, the potential efficacy of STP as monotherapy or in alternative combinations may offer an important therapeutic option for patients who face limited access to optimal treatment regimens.

### Safety profile

Stiripentol has demonstrated a consistent safety and tolerability profile across clinical trials and real-world studies, including in patients followed for a median of 18 years of continuous use. 65 In the pivotal randomized controlled trials, 66 the most common adverse events (AEs), defined as occurring in > 10% of patients and at least 5% more frequently than with placebo, included somnolence (67% versus 23% with placebo), decreased appetite (46% versus 10%), agitation (27% versus 16%), weight loss (27% versus 6%), and hypotonia (18% versus 13%). Other frequently reported AEs included nausea (15% versus 3%), tremor (15% versus 10%), dysarthria (12% versus 0%), and insomnia (12% versus 7%). Notably, these events were generally mild or moderate in intensity and can be effectively managed through dose adjustments of STP or concomitant ASMs (valproate and clobazam). 5 35 53 58

Importantly, other therapies for DS, such as fenfluramine and cannabidiol, exhibited similar rates of AEs during randomized clinical trials. 36 58 67 68 69 However, discontinuations due to adverse effects were significantly less frequent with STP compared with fenfluramine and the higher dose of cannabidiol (20 mg), 42 reinforcing its favorable tolerability profile.

Stiripentol is known to inhibit the cytochrome P450-mediated metabolism of clobazam, particularly the conversion of its active metabolite, norclobazam, into hydroxy-norclobazam. This interaction leads to increased plasma concentrations of clobazam and its active metabolites, 70 potentially contributing to adverse effects such as somnolence, irritability, or hypotonia. 71 72 Consequently, clobazam dose reduction is often necessary when used concomitantly with STP. Valproate, another common co-medication, may also be affected by modest pharmacokinetic interactions with STP, which could lead to loss of appetite or body weight. 71 72 Therapeutic drug monitoring may assist in guiding appropriate dose adjustments. 65 73 74

Real-world observational studies have confirmed the AE profile observed in randomized controlled trials, with no new safety signals identified. 63 73 75 76 77 78 79 80 81 Moreover, the types and frequencies of AEs in patients who initiated STP before 2 years of age were similar to those reported in older children. 40 Discontinuation due to AEs was relatively infrequent: 6% in pivotal randomized controlled trials and ranging from 0 to 12% in real-world studies, with somnolence, appetite or weight loss, ataxia, and behavioral changes being the most commonly cited causes. 56 63 66 73 76 77 78

## ECONOMIC VALUE OF STP

In addition to its clinical efficacy, STP demonstrates favorable economic value in the treatment of DS. In a German retrospective study of 13 patients with DS, Strzelczyk et al. 82 evaluated the effects of adding STP and clobazam to existing ASM. While the total direct healthcare costs increased by 42.7% following STP and clobazam introduction—driven primarily by the annual cost of STP (€6,610)—there was a marked 39.2% (€2,587) decrease in non-ASM direct costs, especially due to a reduction in inpatient admissions and associated hospital costs. The authors concluded that the higher costs of STP were effectively offset by decreased resource use elsewhere, particularly emergency hospitalizations and inpatient care.

Stiripentol also demonstrates economic value when compared with other DS-specific add-on therapies, such as fenfluramine and cannabidiol. A recent pharmacoeconomic analysis evaluated the cost to achieve predefined levels of seizure reduction or freedom with stiripentol, fenfluramine, and cannabidiol in the US. 83 The analysis used number needed to treat (NNT) estimates derived from a network meta-analysis of randomized controlled trials comparing each drug plus standard care to placebo. 42 Numbers needed to treat were calculated for 3 key outcomes: ≥ 50% reduction in monthly *convulsive* seizure frequency, ≥ 75% reduction, and complete seizure freedom (100%).

Stiripentol demonstrated superior or comparable efficacy across all outcomes. For seizure freedom, STP had an NNT of 3, statistically lower than fenfluramine (NNT = 10) and cannabidiol (NNT = 25), indicating that fewer patients need to be treated with STP to achieve this outcome. For ≥ 50% and ≥ 75% seizure reduction, STP also performed favorably (NNT = 2 for both), compared with fenfluramine (NNT = 2 and 3, respectively) and cannabidiol (NNT = 6 and 10). As a result, the annual cost per patient reaching seizure control was consistently lower with STP.

When annual drug costs were calculated based on average patient weight and approved dosing regimens, STP consistently showed the lowest cost per outcome achieved. For adults, the cost for one patient to achieve seizure freedom was $195,673 with STP, versus $644,681 with cannabidiol and $1,124,784 with fenfluramine. Even at lower response thresholds (≥ 50% or ≥ 75% seizure reduction), STP remained the most cost-efficient option.

Further supporting the economic value of STP, Weston et al. 84 assessed the cost-utility of initiating it versus other licensed add-on therapies (cannabidiol, fenfluramine) or continued background ASM therapy in patients with DS from age 2 years. Using a Markov model with six health states (ranging from “seizure free” to “death”), the analysis estimated quality-adjusted life years (QALYs) and costs over a 15-year time horizon from the perspective of the US healthcare system. Efficacy inputs were derived from indirect comparisons of randomized controlled trials, while utility values and resource use were sourced from DS-specific literature.

Stiripentol generated the highest number of QALYs among all evaluated options and was the most cost-effective strategy under the commonly accepted US willingness-to-pay threshold of $200,000 per QALY. Its incremental cost-effectiveness ratio was estimated at $103,923/QALY versus cannabidiol and $89,110/QALY versus background ASMs. In addition, STP economically dominated fenfluramine, providing more health benefit (+ 0.13 QALYs) at a lower cost (–$119,645). These results remained robust across multiple sensitivity and scenario analyses, including variations in age at treatment initiation (as early as 6 months or in adulthood), treatment duration, dosing intensity, and analytical perspective (healthcare versus societal). Although these data are based on the US setting, the implications are relevant to Brazil, where healthcare resources are limited and reimbursement decisions carefully weigh clinical benefit and cost-effectiveness. 85 In this context, treatments like stiripentol, which offers strong clinical efficacy and favorable economic value, may represent a sustainable option for the management of DS.

In conclusion, DS is a severe and highly refractory developmental and epileptic encephalopathy associated with a significant and multifaceted burden—clinically, developmentally, emotionally, and economically. Its management requires early, sustained, and effective seizure control to mitigate complications such as SE and SUDEP, while also aiming to improve QoL for patients and caregivers.

Stiripentol has consistently demonstrated strong anti-seizure efficacy in randomized controlled trials, real-world studies, and meta-analyses. It reduces the frequency and duration of seizures, increases seizure-free intervals, and has shown effectiveness in preventing and interrupting SE. These clinical benefits extend to very young patients, as STP is currently the only DS-specific treatment approved for use from 6 months of age in the US, and with no minimum age restriction in several other countries. In parallel, economic analyses suggest that STP is a cost-effective therapeutic option compared with other approved add-on therapies, achieving meaningful clinical outcomes at a lower cost.

Taken together, the available clinical and economic evidence supports the role of STP as a key therapeutic option in the management of DS, particularly in the context of early diagnosis and the need for ongoing seizure control. Given the high burden of disease, limited treatment options, and resource constraints in Brazil, ensuring access to therapies like stiripentol may contribute to more equitable and effective care for individuals living with DS in the country.
